# A few words of caution on blood purification in sepsis

**DOI:** 10.1186/s13054-025-05268-z

**Published:** 2025-01-25

**Authors:** Klaus Stahl, Pedro David Wendel-Garcia, Christian Bode, Sascha David

**Affiliations:** 1 Department of Gastroenterology, Hepatology, Infectious Diseases and Endocrinology, Hannover Medical School, Carl-Neuberg Strasse 1, 30625 Hannover, Germany; 2 Division of Cardiothoracic and Vascular Anaesthesia and Intensive Care Medicine, Department of Anaesthesia, General Intensive Care and Pain Management, Medical University of Vienna, Vienna, Austria; 3 Institute of Intensive Care Medicine, University Hospital Zurich, Zurich, Switzerland; 4 Department of Anaesthesiology and Intensive Care Medicine, University Hospital Bonn, Bonn, Germany; 5 Department of Nephrology, Hannover Medical School, Hannover, Germany

Dear Editor,

With great interest we read the recent review by *Bottari *et al*.* describing various technologies and concepts of extracorporeal blood purification (EBP) in patients with sepsis [1]. We would like to congratulate the authors for their thorough and balanced review of the challenges associated with EBP in sepsis. However, it must be underlined that the clinical use of EBP remains complex and is not yet supported by robust evidence. We believe that the current review, by presenting concrete suggestions for routine clinical application, is partly too optimistic and could potentially lead to overuse and inadvertent harm. We have selected a few examples that may serve as a basis for further discussion:

While we agree with the overall conclusions of this review, we respectfully disagree with the *“considerations for current clinical practice”* proposed by our esteemed colleagues. Specifically, given the concerning signals of harm from recent studies [2, 3] and the significant knowledge gaps related to optimal timing, dosing, immune monitoring and compartmentalization, we believe that EBP should not be used in routine clinical practice. Instead, it should remain restricted to clinical trials. How can we justify personalizing treatment when we currently lack the essential understanding needed to determine how this could be effectively achieved?

The authors further suggest rather arbitrarily chosen cut-offs of vasopressor support, SOFA score, lactate, and Interleukin (IL)-6 concentrations for considering cytokine hemoadsorption in patients with sepsis. Their suggested cut-off values are retrieved from uncontrolled retrospective registers and observational studies but not from randomized trials. Realistically, these suggested cut-offs apply to many patients with septic shock and, therefore, do not warrant the term “personalized medicine”. In a larger propensity-score-matched analysis suggesting improved outcomes with cytokine adsorption, the patients exhibited severe refractory shock indicated by significant higher vasopressor doses (mean norepinephrine 0.48 ug/kg/min) and lactate concentrations (mean 6.4 mmol/l) at study inclusion [4]. Comparable high shock severity was also found in two prospective studies demonstrating rapid hemodynamic stabilization following TPE [5, 6]. Interestingly, in these studies, especially lactate concentrations at baseline were strong predictors of subsequent treatment response [7]. Nevertheless, patient selection by means of biomarkers for EBP remains more than elusive [3], and future research on predicting treatment response to extracorporeal blood purification therapies in septic shock should focus on exploring patient-specific approaches, such as biomarker-driven identification of inflammatory or coagulopathic [8, 9] sepsis phenotypes.

Another concerning suggestion by Bottari et al. is the proposed sorbent change interval of 12–24 h, for which we believe there is currently no supporting data. In fact, a recent “Matters Arising” article in *Critical Care* highlighted the phenomenon of desorption of target molecules from saturated adsorbers [10]. While this may not be relevant for patients with an IL-6 level of 500 pg/ml (as suggested for inclusion), it could be of concern for those with IL-6 levels in the tens of thousands.

A further aspect worth commenting on is the combination of therapeutic plasma exchange (TPE) together with CPFA, i.e. coupled plasma filtration and adsorption in this review. The two technologies share little in common apart from the term “plasma”. Whereas CPFA is a pure removal strategy (that was also proven harmful in a prematurely stopped RCT) [2], TPE is based on a fundamentally different theoretical approach [11]. While diseased patients´ plasma is not only removed, but substituted to the same extent with healthy donors` plasma, this allows for correction of acquired deficiencies of multiple, potentially protective mediators of the septic syndrome, e.g. anti-permeability [5, 12], anti-thrombotic- [12] and glycocalyx-protective [13] factors as well as deficient immunoglobulins [14]. In fact, this dual therapeutic principle of simultaneous removal and replacement, makes TPE truly unique among all other EBP modalities exploratively used in sepsis, highlighting it as the only EBP with randomized evidence suggestive of benefit.

In summary, while we are advocates for blood purification in sepsis, we recognize that relying solely on biological plausibility has, on numerous occasions, led us astray in the past. As Robert Kennedy once said “*Our future may lie beyond our vision*, *but it is not completely beyond our control.*” Multicentric randomized controlled trials, such as the ACYSS (NCT04013269) and the Exchange-2 (NCT05726825) study [15], are pivotal in shaping the future vision of EBP in septic shock.

## Data Availability

No datasets were generated or analysed during the current study.
